# Drinking Water Quality Assessment Using a Fuzzy Inference System Method: A Case Study of Rome (Italy)

**DOI:** 10.3390/ijerph20156522

**Published:** 2023-08-04

**Authors:** Yas Barzegar, Irina Gorelova, Francesco Bellini, Fabrizio D’Ascenzo

**Affiliations:** Department of Management, Sapienza University of Rome, 00161 Rome, Italy; yas.barzegar@uniroma1.it (Y.B.); francesco.bellini@uniroma1.it (F.B.); fabrizio.dascenzo@uniroma1.it (F.D.)

**Keywords:** water quality, drinking water, fuzzy logic, fuzzy inference systems, membership functions, water attribute, smart city

## Abstract

Drinking water quality assessment is a major issue today, as it is crucial to supply safe drinking water to ensure the well-being of society. Predicting drinking water quality helps strengthen water management and fight water pollution; technologies and practices for drinking water quality assessment are continuously improving; artificial intelligence methods prove their efficiency in this domain. This research effort seeks a hierarchical fuzzy model for predicting drinking water quality in Rome (Italy). The Mamdani fuzzy inference system is applied with different defuzzification methods. The proposed model includes three fuzzy intermediate models and one fuzzy final model. Each model consists of three input parameters and 27 fuzzy rules. A water quality assessment model is developed with a dataset that considers nine parameters (alkalinity, hardness, pH, Ca, Mg, fluoride, sulphate, nitrates, and iron). These nine parameters of drinking water are anticipated to be within the acceptable limits set to protect human health. Fuzzy-logic-based methods have been demonstrated to be appropriate to address uncertainty and subjectivity in drinking water quality assessment; they are an effective method for managing complicated, uncertain water systems and predicting drinking water quality. The proposed method can provide an effective solution for complex systems; this method can be modified easily to improve performance.

## 1. Introduction

Water quality issues are crucially important for the well-being of world society; Sustainable Development Goal 6 of the United Nations (UN) states the importance of ensuring the availability and sustainable management of water and sanitation for all [1]. According to the UN Sustainable Development Goals Report (2022), the average global implementation rate of enhanced water resources management must double to guarantee a sustainable and fair water allocation that would suit all demands [2]. The UN Sustainable Development Goals in general, and the well-being of people and the planet, depend on water. However, the worldwide progress toward water-related objectives and targets still appears to be significantly off course, affecting the overall agenda for sustainable development. There is, therefore, an obvious need for a Water Action Agenda as a result of the UN 2023 Water Conference; the UN emphasizes the need for unifying international society as to actions that help to deliver water-related scalable activities that can be replicated in the future. These commitments, pledges, and actions must cut across our sectors, industries, and interests. A “beyond business as usual” strategy will be required to address the present and future difficulties in the water domain [3].

Water quality assessment and management plays a pivotal role in ensuring the health and well-being of the population, protecting biological ecosystems, and supporting sustainable development in urban and rural environments [4,5]. Water quality assessment is especially important for monitoring and ensuring the safety of drinking water, as regular monitoring and testing allow for the implementation of appropriate treatment and purification processes to provide clean and safe drinking water for the population [6]. Water quality modeling and prediction have become extremely significant in the control of water pollution, environmental protection, and the decrease of water-related disorders [7,8,9]. Water quality assessment is critical for building sustainable and livable environments; advanced technologies for analyzing and monitoring water quality in real-time provide better living conditions to the population and help societies respond promptly to emergencies [10,11]. The importance of drinking water safety has arisen in the context of the COVID-19 pandemic outbreak, as the COVID-19 virus can persist in untreated drinking water; however, water quality assessment and treatment methods may significantly reduce the concentration of SARS-CoV-2 and other viruses in water [12].

Scholars are continuously looking for solutions for effective drinking water quality assessment. Traditional methods of measuring and estimating drinking water quality usually involve a combination of physical, chemical, and microbiological tests, for example, turbidity tests [13,14], total dissolved solids tests, and total suspended matter tests [15,16,17], in addition to pH measurement [18,19]; these methods are especially employed in areas with limited technological infrastructure, and lacking advanced equipment and methodologies which would offer more comprehensive and accurate analyses of drinking water quality. The scientific literature evidences effective nanotechnology methods used in the measurement of major cations, anions, and heavy metals in water [20,21]. Artificial intelligence (AI) technologies for drinking water quality assessment are represented in the scientific literature by different methods, for example, adaptive neuro-fuzzy inference systems [22], artificial neural network models [22,23,24], machine learning (ML) approaches [25,26,27,28,29,30], and fuzzy inference systems (FIS) [31]. The robustness of the FIS method applied to this research is due to its simplicity in dealing with uncertainty and its immediate assessment of concentrations and values of different water quality parameters that integrate to define a water ecosystem.

Providing clean and safe water for its inhabitants and conserving water are crucial aspects of a sustainable and livable urban environment, especially in a smart city [32,33,34]. The present research provides a case study of the Municipality of Rome (Italy), which presented the Rome Smart City strategic plan in 2020 [35]. The Rome Smart City plan aims to create a methodological and strategic framework to bring together the priority objectives in the various areas of Rome, such as mobility, environment and waste, social, culture, tourism, safety, etc. The plan is intended to be a dynamic tool that incorporates the needs and expectations of the city users and sees citizens as protagonists of the co-creation process. Among other domains, the Rome Smart City plan focuses on environmental sustainability in terms of the reduction of pollution and the improvement of air quality, together with waste management and the water cycle. A number of sensors for leak monitoring of water, a number of sensors for quality monitoring of the water, and a number of water purification plants are among the Smart Key Performance Indicators (KPIs) of the Rome Smart City plan, indicators which represent the level of digitalization of the city and the use of innovative technologies to provide services and instruments for the improvement of the urban environment. Implementation of these water quality assessment measures in the municipality of Rome aligns with supranational and national regulations [36,37,38].

This study aims to model a system based on the FIS method to predict the drinking water quality based on the data from the city of Rome and to demonstrate the application of the model to the identification of the importance of the variables applied. The goals of the research are to understand: (1) whether the FIS method is suitable for addressing environmental issues involving ambiguity and uncertainty, and especially to assess the quality of different types of water; (2) if the FIS method helps to understand and evaluate the effect of each input parameter on the final water quality, and (3) if the FIS method can apply different types of defuzzification methods and membership functions.

## 2. Materials and Methods

### 2.1. Research Area and Dataset for the Water Quality Predictive Model

In this present study, the data was taken from the open database of the ACEA ELABORI group [39]. The data include some important physical and chemical properties of the drinking water distributed in Rome in 2018. The sampling area includes different areas such as distribution networks, with 320 sampling points, water system and water feed pipes, with 26 sampling points, and water centers, with 21 sampling points in Rome. ACEA ELABORI group, the leading Italian operator in the sector of integrated water services by the number of residents served, operates in five Italian regions: Lazio, Tuscany, Umbria, Molise, and Campania. They manage the entire water cycle, from the spring to wastewater treatment, for nine million residents in five Italian regions. In Rome, the qualitative characteristics of the resources collected and distributed are monitored through continuous testing with instruments located along the water systems and through daily sampling at the collectors and in the distribution network.

### 2.2. Fuzzy Inference System

In this research, we applied a FIS model, the mathematical technique of employing fuzzy logic to map a given input to an output. The fuzzy inference process consists of three crucial components: fuzzification, if–then rules, and defuzzification [40]. Due to ambiguity and vagueness in the water quality data, a fuzzy inference approach can be suitable for resolving ambiguity and evaluating water quality [41]. The most popular fuzzy inference technique is called the Mamdani method. Zadeh presented the fuzzy logic model in 1965 [42].

#### 2.2.1. Operation of Fuzzy Inference System

**Figure 1:**
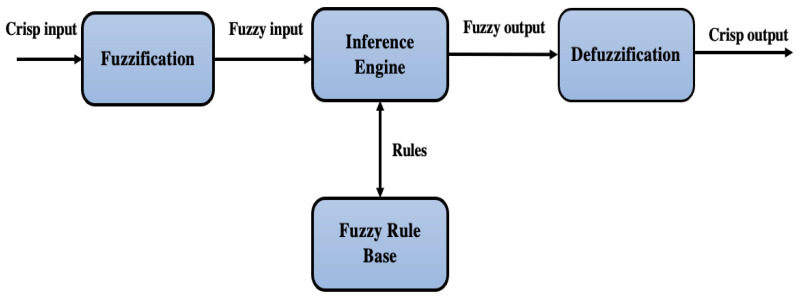
Basic structure of a fuzzy inference system.

The robust Mamdani fuzzy inference system consists of four main steps: (a) fuzzification of variables, (b) rule evaluation, (c) aggregation of the rule, and (d) defuzzification. Figure 1 represents the basic structure of the FIS.

#### 2.2.2. Fuzzification

The first stage of the FIS is fuzzification. In this stage, crisp inputs are transformed into fuzzy inputs known as linguistic variables through different membership functions. A membership function is a graphical representation that quantifies linguistic terms and represents a fuzzy set graphically [43]. Common membership functions are triangular, Gaussian, trapezoidal, and bell-curved. Therefore, fuzzification aims to assign the numerical input values to membership grades in fuzzy sets specified with text. In this study, we applied a triangular membership function for both input and output parameters. The triangular membership function is one of the most widely accepted and has the advantages of simplicity and used membership functions (MF) in fuzzy controller design. The triangle that fuzzifies the input can be defined by three parameters, a, b, and c, where c defines the base and b defines the height of the triangle.

#### 2.2.3. Inference Engine

The inference engine uses the knowledge base’s fuzzy rules to generate the fuzzy output. This output cannot be used directly in any processes or systems, so it has to be converted into a crisp output. A fuzzy rule is an expression with conditions defined as if–then. The fuzzy rule is the following: If X is A, then Y is B. In this fuzzy rule, X and Y are linguistic variables, and A and B are fuzzy sets. After defining all of the If–then rules, the aggregation process is applied to combine all the rules to obtain one single fuzzy set.

#### 2.2.4. Defuzzification

The defuzzifier is a critical component of an expert system. Defuzzification is the final stage in fuzzy inference system processing [44]. It is the process of converting the fuzzy input to the crisp output [45]. The defuzzifier is classified into several types, which include the mean of maximum method (MOM), the centroid of area (COA), the largest of maximum (LOM), the bisector of area (BOA), and the smallest of maximum (SOM). The center of area (CoA) defuzzification method, which is the most common defuzzification method, is also called the center of gravity (CoG) method. In this defuzzification method (CoA), first, the fuzzy controller calculates the area under the scaled membership functions and within the range of the output variable. Then the fuzzy logic controller uses the following equation to calculate the geometric center of this area, where CoA is the center of the area, x is the value of the linguistic variable, and x_min_ and x_max_ represent the range of the linguistic variable.

(1) $$COA=\frac{\int_{X_{min}}^{X_{max}}f\left(x\right)*x}{\int_{X_{min}}^{X_{max}}f(x)}$$

### 2.3. Development of a FIS Model for Prediction of Drinking Water Quality Distributed in Rome

**Figure 2:**
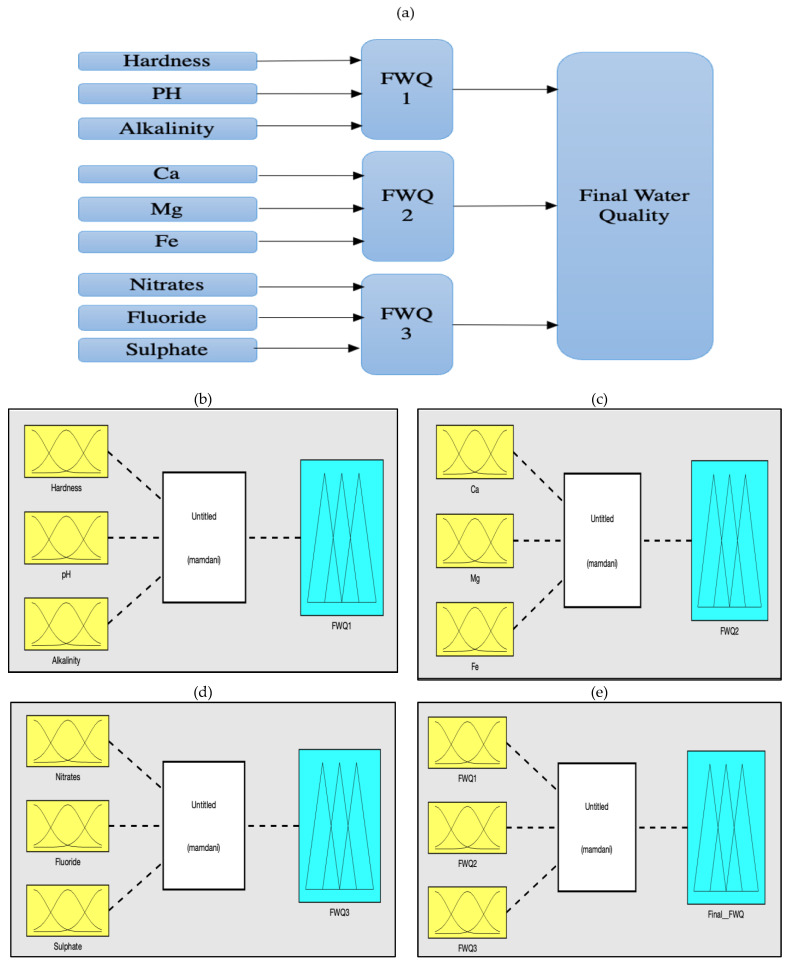
(**a**) Structure of hierarchical fuzzy model for prediction of water quality; fuzzy models in MATLAB software. (**b**) First fuzzy model. (**c**) Second fuzzy model. (**d**) Third fuzzy model. (**e**) Final fuzzy model.

The current research project seeks to create a hierarchical fuzzy model for predicting water quality. This model for water quality prediction has been implemented on Matlab R2022b software (MathWorks, Natick, Massachusetts, MA, USA). Figure 2a presents the structure of a hierarchical fuzzy inference model for predicting drinking water quality. The methodology for developing the fuzzy model to predict water quality involves several steps. The first step is determining the system’s input and output variables. The structure of the fuzzy model shows that the first fuzzy model (FWQ1) has three inputs: alkalinity, pH, and hardness. The second fuzzy model (FWQ2) also has three inputs: Ca, Mg, and Fe. The third fuzzy model (FWQ3) has three inputs: sulfate, nitrate, and fluoride. The output of each model (FWQ1, FWQ2, and FWQ3) will be the input of the final model to obtain the final output of the water quality assessment. Figure 2b–e show the structure of three intermediate fuzzy water-quality models and a final fuzzy water-quality model in Matlab Software (MathWorks, Natick, Massachusetts, MA, USA).

After defining the inputs and outputs of the model, the next step is to determine ranges for each input and output. The range for each parameter is determined based on the drinking water quality standards (IS 10500) which are listed in Table 1. The amount and presence of physicochemical and biological parameters of drinking water set by regulatory bodies are anticipated to be within the acceptable limits (Desirable Limit) to protect human health. Parameters within this allowed range provide no health risk to water users, while deviation from the limit in excess of the tolerable level results in a human health disaster [46].

The numerical values of all inputs of the model are divided into three categories, which are high, medium, and low, and the output of the proposed model is divided into seven categories between 0-100 which are: very, very low (VVL) (0–16.67), very low (VL) (0–33.3), low (16.67–50), medium(M) (33.33–66.67), high(H) (50–83.3), very high (VH) (66.67–100), and very, very high (VVH) (83.33–100). The description of all input variables with their linguistic variables and their ranges is shown in Table 1.

**Figure 3:**
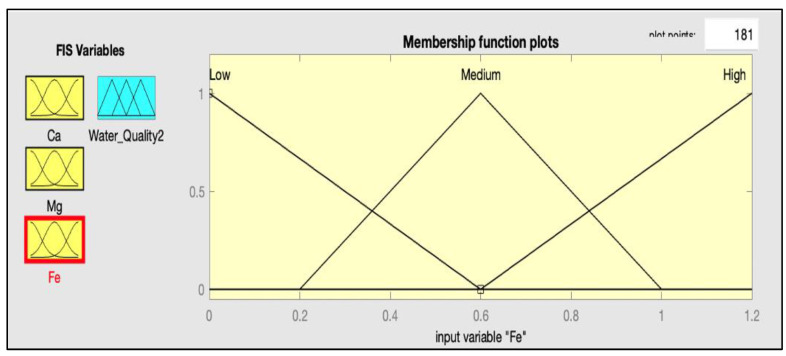
Membership function for Fe.

**Figure 4:**
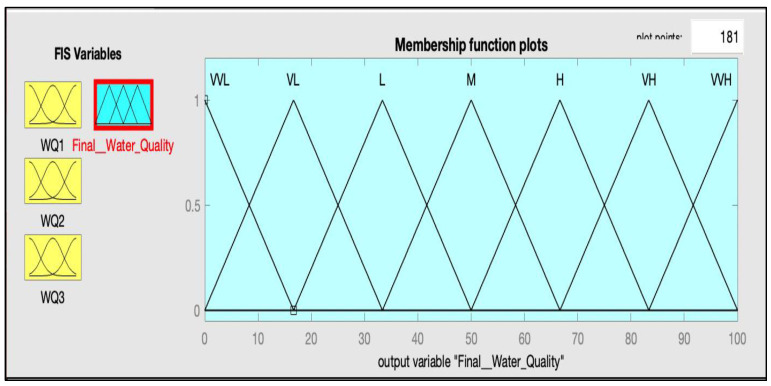
Membership function for output.

After defining the range for each parameter, the next step is the selection of the membership function for each input and output. The FIS is used to fuzzify the crisp input; in this way, the fuzzy set transforms to a crisp number. A membership function (MF) is a curve that specifies how each point in the input space is assigned to a membership value. In Figure 3 and Figure 4, the MFs for some input variables and output variables are presented.

**Figure 5:**
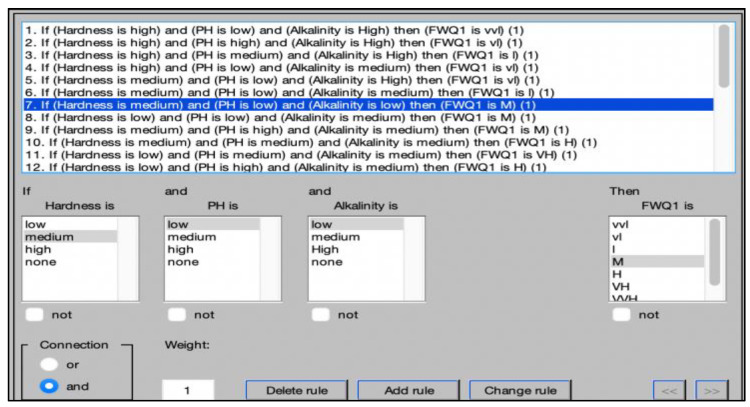
Fuzzy rules for first fuzzy model.

The fourth step is to write the linguistic rules. The rule base is a collection of linguistic statements in the form of if–then rules, which include antecedents and consequences linked by the “and” operator. Figure 5 presents the if–then rules for the FWQ1 and FWQ2 models, respectively. After all of the if–then rules are defined, all the fuzzy rules are evaluated in the inference engine and then aggregated to obtain one single rule, and, in the final step, the defuzzification method is applied to convert the fuzzy output into a crisp output.

## 3. Results and Discussion

**Figure 6:**
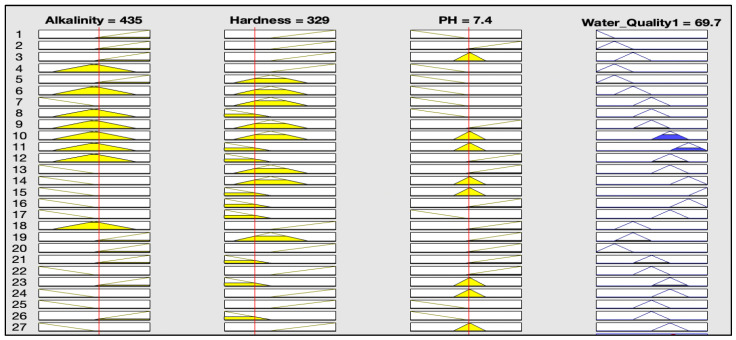
Water quality for the first model, with centroid defuzzification.

**Figure 7:**
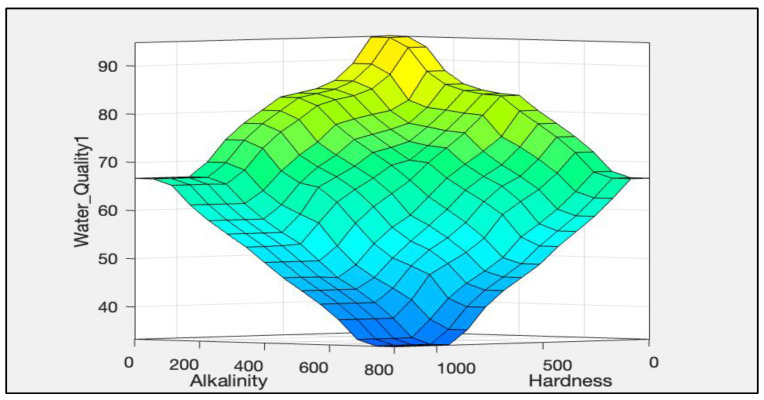
Surface viewer of first fuzzy model.

**Figure 8:**
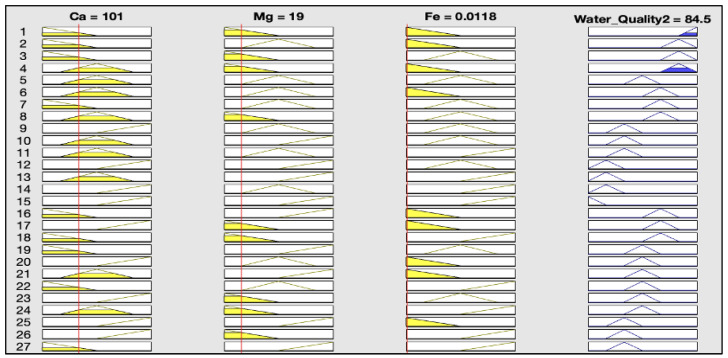
Rule viewer of the second model, with centroid defuzzification method.

**Figure 9:**
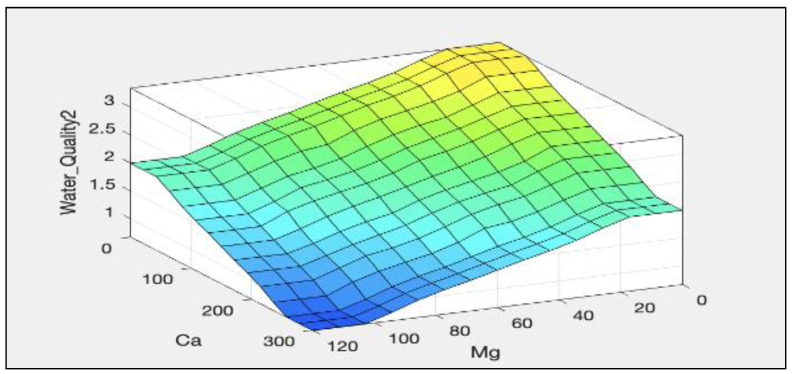
Surface viewer of the second model, with centroid defuzzification method.

**Figure 10:**
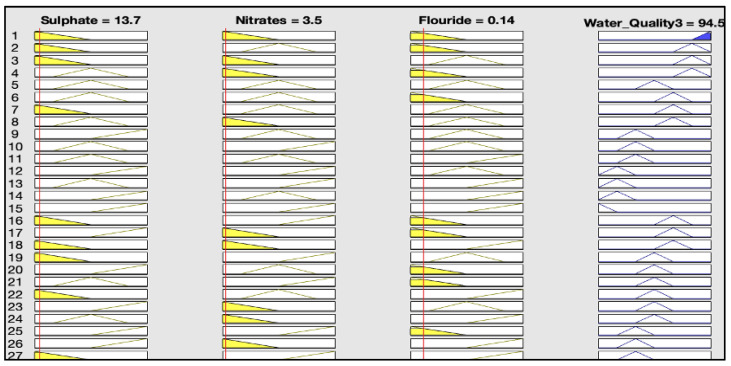
Rule viewer for water quality assessment in the third model.

**Figure 11:**
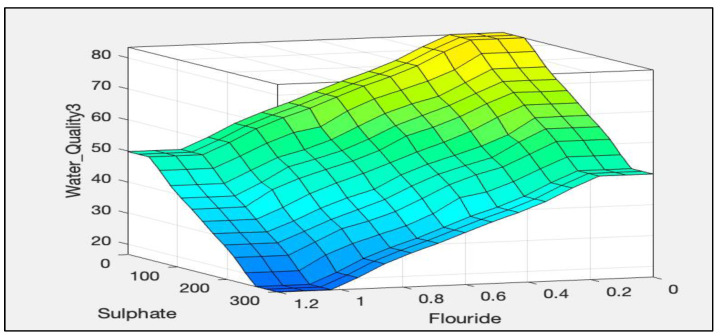
Surface viewer of water quality in the third model.

**Figure 12:**
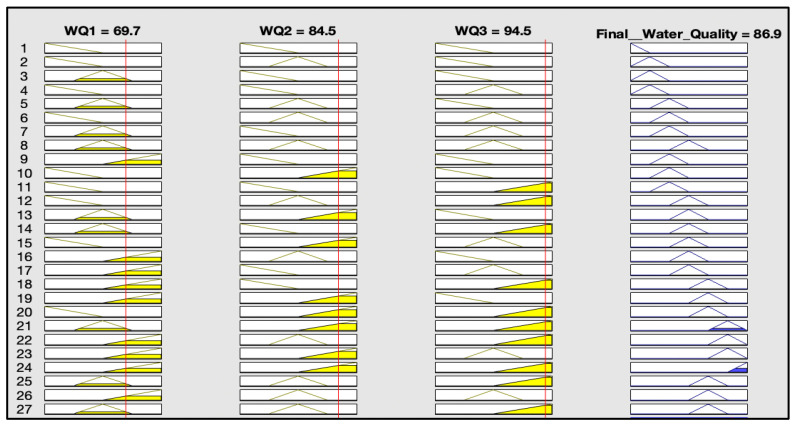
Rule viewer for final water quality assessment, with centroid defuzzification.

The rules representations of the four models, FWQ1, FWQ2, FWQ3, and the final FWQ, all with a centroid defuzzification method, are shown in Figures 6, 8, 10, and 12, respectively. Figures 7, 9, 11, and 14 illustrate surface views of the FWQ1, FWQ2, FWQ3, and FWQ models, respectively. Different types of defuzzification techniques are used in this study.

### 3.1. Intermediate Models

#### 3.1.1. First Fuzzy Model (FWQ1)

Figure 6 shows the rule viewer for water quality assessment in the first model (FWQ1) with three input parameters: hardness, alkalinity, and pH. The rule-base representation in Figure 6 indicates that the value for water quality in the FWQ1 is 69.7%, which is obtained with the centroid defuzzification method for the respective average concentrations or values of alkalinity, pH, and hardness, which are 435, 7.4, and 329 mg/L, respectively.

The rule-base representation in Table 2 indicates that the value of water quality for the FWQ1 is 60% with the SOM defuzzification method, 70% with bisector defuzzification and 66.5% with MOM defuzzification, respectively, for the respective average concentrations or values of alkalinity, pH, and hardness, which are 435, 7.4, and 329 mg/L, respectively.

Figure 7 shows the effects of hardness and alkalinity on water quality in FWQ1; it illustrates that the lower the hardness and alkalinity values are, the higher is the water quality. Figure 7 also demonstrates that as the concentration of alkalinity and hardness increased, the value of water quality decreased, and vice versa. Figure 7 shows FWQ1 values as a function of hardness and alkalinity as the inputs, while the third input (pH) is hidden in this view.

#### 3.1.2. Second Fuzzy Model (FWQ2)

Figure 8 shows the rule viewer for water quality assessment in the second model (FWQ2), with three input parameters: Ca, Mg, and Fe. The rule-base representation in Figure 8 indicates that the value for water quality in the FWQ2 is 84.5%, which is obtained by the centroid defuzzification method for the respective average concentrations of Ca, Mg, and Fe, which are 101, 19, and 0.0118 mg/L, respectively.

Figure 9 shows the effects of Ca and Mg on water quality; the lower the Ca and Mg concentrations are, the higher is the value for water quality. Figure 9 demonstrates the water quality value in the FWQ2 as a function of Ca and Mg concentration as the inputs, while the third input (Fe) is hidden in this view; as the concentrations of Ca and Mg increased, the value of water quality decreased, and vice versa.

Table 3 indicates the water quality assessment in different conditions with different concentrations of Ca, Fe and Mg, with the centroid defuzzification method used to evaluate the effect of each input parameter on the water quality assessment. Table 3 shows that as the concentrations of Ca, Mg, and Fe increased, the value of water quality decreased, and vice versa.

#### 3.1.3. Third Fuzzy Model (FWQ3)

Figure 10 shows the rule viewer for water quality assessment in the third model (FWQ3), with three input parameters: sulfate, nitrate, and fluoride. The rule-base representation in Figure 10 indicates that the value of FWQ3 is 94.5% with the centroid defuzzification method for the respective average concentrations of sulfate, nitrate, and fluoride, which are 13.7, 3.5, and 0.14 mg/L, respectively.

Table 4 shows the rule viewer for water quality assessment in the FWQ3 with three input parameters: sulfate, nitrate, and fluoride, by different defuzzification methods. The rule-base representation in Table 4 indicates that the value of FWQ3 is 95% with the bisector defuzzification method, 97% with the SOM defuzzification method, and 98.5% with the MOM defuzzification method for the respective average concentrations of sulfate, nitrate, and fluoride, which are 13.7, 3.5, and 0.14 mg/L, respectively.

Figure 11 shows the effects of sulphate, fluoride, and nitrates on water quality; the lower the sulphate, fluoride, and nitrates values are, the higher is the water quality.

Table 5 represents the results of the water quality assessment with different amounts of sulphate, nitrate, and fluoride by the centroid defuzzification method, which was used to evaluate the effect of each parameter on the quality of the water. According to Table 5, the water quality is a function of the amount of sulphate, nitrate, and fluoride. As the amounts of sulphate, nitrate, and fluoride increased, the value of water quality decreased, and vice versa.

### 3.2. Final Model (FWQ)

Figure 12 shows the rule viewer for water quality assessment in the FWQ with three input parameters, which are FWQ1, FWQ2, and FWQ3. The rule-base representation in Figure 12 indicates that the value of Final FWQ is 86.9% for the respective average values of FWQ1, FWQ2, and FWQ3, which are 69.7, 84.5, and 94.5%, respectively, with centroid defuzzification. It shows that the FWQ with the centroid defuzzification method is very high.

**Figure 13:**
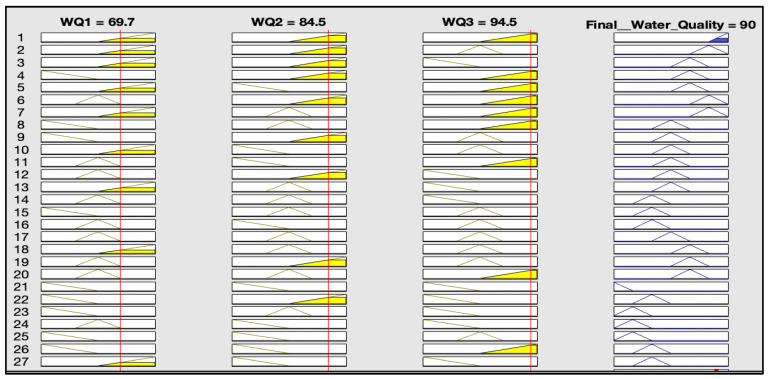
Final water quality assessment with SOM defuzzification.

Figure 13 shows the rule viewer for water quality assessment in the FWQ with three input parameters, which are FWQ1, FWQ2, and FWQ3. The rule-base representation in Figure 13 indicates that the value of FWQ is 90% for the respective average values of FWQ1, FWQ2 and FWQ3, which are 69.7, 84.5, and 94.5%, respectively, with the SOM defuzzification method.

Table 6 shows the final value of water quality in the last model in different situations with different percentages of input parameters. The table shows each parameter’s effect on the water quality value. According to the table, the water quality is a function of all the proposed fuzzy model input parameters.

**Figure 14:**
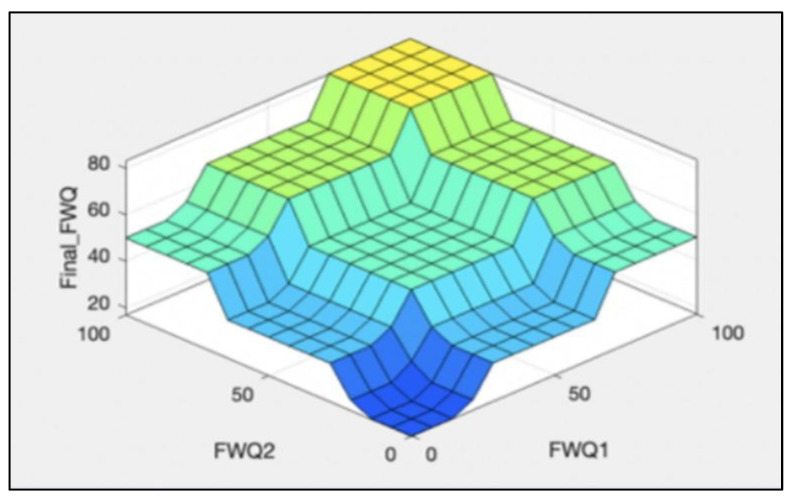
Surface viewer for final water quality.

Figure 14 shows the effects of FWQ1 and FWQ2 values on the final value of water quality. It shows that the higher are the FWQ1 and FWQ2 values, the higher is the final value of water quality. As the amounts of FWQ1and FWQ2 increased, the value of final water quality increased, and vice versa.

### 3.3. Validation of the Model

In order to validate the water quality model, we compared the predicted value of the final fuzzy water quality index (WQI) from the model with that of the deterministic values of WQI. The formula used to determine the aggregated water quality index is given in Equation (2), where, I_i_ is the sub-index of the ith water quality parameter, WQI is water quality index, and ‘n’ is the number of water quality parameters considered. W_i_ is the weightage of the ith water quality parameter, The formula used to determine the aggregated water quality index is given in Equation (2).

(2) $$WQI=\sum_{i=1}^{n}W_{i}I_{i}$$

The sub-index of the ith quality parameter can be determined by Equation (3).

(3) $$I=\frac{C_{s}-C_{i}}{C_{s}-C_{min}}$$

where C_i_ is the observed concentration of the ith water quality parameter, Cs the concentration limit value of the ith water quality parameter, as mentioned in Table 1, and C_min_ the minimum concentration of the parameter reflecting the best water quality. The minimum values for all the parameters considered in the model are 0, except for pH (pH = 7 represents the best water quality).

**Figure 15:**
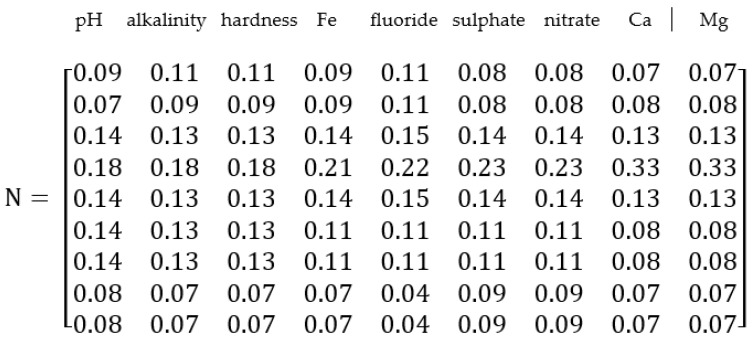
The normalized matrix is represented in normalized comparison matrix N.

The weightage of individual pollutants can be found out using an analytical hierarchy process (AHP). AHP is a systematic method for comparing a list of objectives or alternatives. This method forms a pair-wise comparison matrix. The comparison matrix is generated by expert ranking using Saaty’s scale (1980) [47]. We normalized the comparison matrix by taking the sum of each column and then divided each column by the corresponding sum to obtain the normalized matrix. The normalized matrix thus obtained is represented in normalized comparison matrix N (Figure 15).

**Figure 16:**
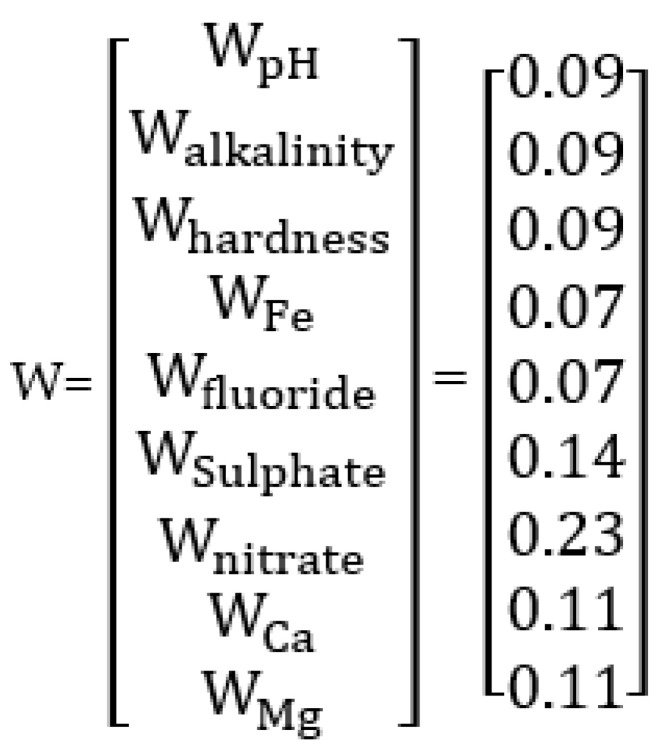
The relative weight vector W for the pollutants.

The relative weight vector W for the pollutants is given by the average of the row elements in comparison matrix (Figure 16), as:

Table 7 shows the comparison of the final result of the water quality model with the fuzzy inference water quality and water quality index. The result of our model with a fuzzy inference system shows the percentage for water quality with the centroid defuzzification method as 86.9% and with the water quality index approach, it shows the percentage for water quality as 77%.

### 3.4. Discussion

In this study, the FIS approach was used to build the Mamdani fuzzy water-quality inference engine, which is known for its simple structure and max–min inference. The computational tool used in modeling the overall system was the Matlab Fuzzy Logic. The implication method used in this approach was the “min”, the aggregation method was “max”, and for the defuzzification method, we used different types of methods, such as MOM, COA, LOM, BOA, and SOM. The result of this research on water quality assessment, which used a FIS method, shows that the final water quality measurement is a function of each of nine input parameters. A water quality measurement expresses the overall water quality in a given place and time based on different physical and chemical parameters. We divided the output values into seven classes, as shown in Figure 4. Overall, seven fuzzy sets, which are “very, very high”, “very high”, “high”, “medium”, “low”, “very low”, and “very, very low”, were considered for this study, both for input indicators and the output value of water quality.

There are several studies using different types of AI in the field of water quality measurement. The robustness of FIS methodology in comparison with other AI methods is in its simplicity and ability to deal with uncertainty, as well as its immediate assessment of concentrations and values of different water quality parameters that integrate a water ecosystem. FIS has a reasoning process to handle uncertainty and subjectivity. The neural networks and particle swarm algorithms were applied for prediction of the quality parameters of the Yang River [48]; the results of the study showed that the particle swarm algorithm had a desirable effect on the operation of neural networks to predict the quality parameters of river water. In another study [49],the parameters of dissolved oxygen (DO) and chemical oxygen demand (COD) were modeled in India’s Mehi river; the researchers understood that an adaptive neuro-fuzzy inference system (ANFIS) had suitable ability in assessing the mentioned parameters for the Mehi River. Also, the scholars [50] evaluated the ability of trained ANFIS with hybrid algorithms in predicting water resources. Another group of researchers [51] compared two data-driven models, ANFIS and Gaussian process (GP), to predict and simulate water quality parameters, and proposed genetic programming as an effective tool for determining water quality parameters. Other works analyze ecological problems for aspects of environmental pollution by applying AI techniques such as artificial neural networks [52,53], support vector machines [54,55], and factor analysis [56]. All of them lack a reasoning process to handle uncertainty and subjectivity. Compared with traditional machine-learning methods, such as the support vector machine (SVM) and radio frequency (RF), the convolutional neural network (CNN) had the best classification accuracy. The performance of the SVM method as applied to predict coagulant dosage in water treatment plants of distinct sizes was analyzed [57], and the results show that such a method performs better for large- and medium-sized water systems compared to small ones. Although it shares similarities with artificial neural networks (ANNs), SVM shows a better ability to deal with high-dimensional data and is less prone to overfitting [58].

The ML approach helped to identify patterns of association between predictor and response variables based on a data-driven system by extracting knowledge from the historical database. In particular, the data-driven fuzzy approach proved to be efficient in solving this complex and poorly understood problem, a problem characterized by uncertainty due to imprecise knowledge. In other words, data-driven fuzzy analysis allows for dealing with uncertainties and provides a powerful framework for computational reasoning [59]. As a limitation, classical ML methods were not designed to deal with uncertainty. Therefore, when the degree of uncertainty of the problem becomes significant, the solution provided by classical ML methods is not able to provide a solution with greater accuracy [60]. In general, although some ML algorithms stand out for their high performance in specific applications, it is essential to note that task accuracy is also highly associated with data behavior [61]. Therefore, comparing several ML methods is important for verifying the best alternative applicable to each case [62]. The ANFIS suffers from limitations, such as the curse-of-dimensionality computational expense, and it is not good at explaining how it reaches decisions [63].

## 4. Conclusions

This research effort seeks to create a hierarchical fuzzy model for predicting drinking water quality. This study applies the Mamdani fuzzy inference system, but with a different defuzzification method. The proposed model includes three fuzzy intermediate models and one fuzzy final model. Each fuzzy model consists of three input parameters and 27 fuzzy rules. The model is developed for water quality assessment with a dataset considering nine important parameters (alkalinity, hardness, pH, Ca, Mg, fluoride, sulphate, nitrates, and iron). The results of the research show how the concentration of the nine above-mentioned parameters affects the water quality. The result shows that when the pH is in the medium range (5.5–9.5), alkalinity has a low concentration (0–400 mg/L), hardness has a low concentration (0–500 mg/L), Ca has a low concentration (0–150 mg/L), Mg has a low concentration (0–60 mg/L), Fe has a low concentration (0–0.6 mg/L), fluoride has a low concentration (0–3 mg/L), nitrate has a low concentration (0–80 mg/L), sulphate has a low concentration (0–400 mg/L), and the water quality is very, very high.

The FIS is a robust decision-making tool for predicting drinking water quality and calculating water quality with a numeric value, which is easier to understand. This numeric value can help decision makers easily understand the state and situation of drinking water quality and apply the required action if needed. The FIS can provide an effective solution for complex systems, and this method can be easily modified to improve performance. The outcomes of this research are: (1) the FIS method is suitable for addressing environmental issues involving ambiguity and uncertainty, especially assessing water quality in order to overcome the uncertainty of water quality and obtain a crisp output; (2) it is possible to use the FIS method for assessing the quality of the different types of water such as surface water, groundwater, wastewater, and recycled water; (3) the FIS helps us to understand and evaluate the effect of each input parameter on the final water quality, and this result can help the decision makers; (4) since the Mamdani fuzzy inference system is rule-based, it is easier to understand; and (5) the FIS can apply different types of defuzzification methods, and, also, it is possible to apply different types of membership functions. Future studies can focus on applying the ANFIS method, which combines a neural network with a FIS. Also, for future research, it is possible to develop this model, add more input parameters and use other membership functions, such as trapezoidal and Gaussian membership functions, to have a more accurate and complete model.

## Figures and Tables

**Table 1:** Fuzzy value and desirable value for each input of the model.

Parameters	Low	Medium	High	Desirable Limit	Data Set of Rome
pH	0–7 (Poor)	5.5–9.5 (Good)	7–14 (Moderate)	6.5–8.5	7.4
Alkalinity	0–400 (Good)	100–700 (Moderate)	400–800 (Poor)	200 mg/L	435 mg/L
Hardness	0–500 (Good)	100–900 (Moderate)	500–1200(Poor)	300 mg/L	329 mg/L
Ca	0–150 (Good)	50–250 (Moderate)	150–300 (Poor)	75 mg/L	100.6 mg/L
Mg	0–60 (Good)	20–100 (Moderate)	60–120 (Poor)	30 mg/L	19 mg/L
Fe	0–0.6 (Good)	0.2–1 (Moderate)	0.6–1.2 (Poor)	0.3 mg/L	0.0118 mg/L
Fluoride	0–3 (Good)	1–5 (Moderate)	3–6 (Poor)	1.5 mg/L	0.14 mg/L
Nitrates	0–80 (Good)	20–140 (Moderate)	80–180 (Poor)	45 mg/L	3.5 mg/L
Sulphate	0–400 (Good)	150–650 (Moderate)	400–800 (Poor)	200 mg/L	13.7 mg/L

**Table 2:** Water quality in the first model, with different types of defuzzification.

Alkalinity mg/L	Hardness mg/L	pH mg/L	Water Quality 1 with Centroid Defuzzification Method	Water Quality 1 with SOM Defuzzification Method	Water Quality 1 with Bisector Defuzzification Method	Water Quality 1 with MOM Defuzzification Method
435	7.4	329	69.7%	60%	70%	66.5%

**Table 3:** Water quality assessment in different conditions by the centroid defuzzification method.

Ca	Mg	Fe	FWQ2
195 mg/L	23.8 mg/L	0.667 mg/L	53.5%
195 mg/L	23.8 mg/L	0.384 mg/L	64.8%
122 mg/L	23.8 mg/L	0.384 mg/L	72%
122 mg/L	76.5 mg/L	0.867 mg/L	39.2%
122 mg/L	25.2 mg/L	0.867 mg/L	58.7%
37.5 mg/L	25.2 mg/L	0.867 mg/L	70.3%
162 mg/L	66.2 mg/L	0.589 mg/L	45.4%

**Table 4:** Effect of different fuzzification methods on the third model of fuzzy water quality.

Sulfate mg/L	Nitrate mg/L	Fluoride mg/L	FWQ3 with Centroid Defuzzification Method	FWQ3 with SOM Defuzzification Method	FWQ3 with Bisector Defuzzification Method	FWQ3 with MOM Defuzzification Method
13.7	3.5	0.14	94.5%	97%	95%	98.5%

**Table 5:** Water quality assessment with different amounts of sulphate, nitrate, and fluoride.

Sulphate	Nitrate	Fluoride	FWQ3
191 mg/L	77.9 mg/L	0.618 mg/L	37.1%
85.1 mg/L	53 mg/L	0.282 mg/L	70.6%
195 mg/L	92.6 mg/L	0.706 mg/L	30.6%
177 mg/L	85.2 mg/L	0.252 mg/L	49%
85.1 mg/L	77.9 mg/L	0.618 mg/L	51.1%
191 mg/L	58.9 mg/L	0.516 mg/L	48.2%
158 mg/L	58.9 mg/L	0.53 mg/L	51.7%

**Table 6:** Final value of water quality in the last model in different situations.

Situation	Hardness mg/L	pH	Alkalinity mg/L	Ca mg/L	Mg mg/L	Fe mg/L	Fluoride mg/L	Sulphate mg/L	Nitrate mg/L	Water Quality %
Situation 1	399	8.07	266	195	23.8	0.667	0.618	191	77.9	56.5
Situation 2	765	9.26	461	195	23.8	0.384	0.282	85.1	53	63.1
Situation 3	896	11	666	122	23.8	0.384	0.706	195	92.6	35.7
Situation 4	735	3.97	227	122	76.2	0.867	0.252	177	85.2	36.3
Situation 5	604	7.55	344	122	25.2	0.867	0.618	85.1	77.9	62.5
Situation 6	926	10.6	139	375	25.2	0.867	0.516	191	58.9	42.9
Situation 7	882	2.09	588	162	66.2	0.589	0.516	158	58.9	32

**Table 7:** Validation of the model.

pH	Hardness mg/L	Alkalinity mg/L	Fe mg/L	Fluoride mg/L	Sulphate mg/L	Nitrate mg/L	Ca mg/L	Mg mg/L	FWQ %	WQI %
7.4	329	435	0.0118	1.5	13.7	3.5	100.6	19	86.9	77

## Data Availability

Publicly available datasets were analyzed in this study. This data can be found here: (http://sostenibilita2018.gruppo.acea.it/en/relations-environment/water-segment/water-quality, accessed on 10 March 2023).
